# Supplementary material for: Endoplasmic Reticulum Stress Causing Apoptosis in a Mouse Model of an Ischemic Spinal Cord Injury
Source: Int J Mol Sci. 2023 Jan 9;24(2):1307. doi: 10.3390/ijms24021307 (PMC9862494; doi:10.3390/ijms24021307)
Supplement: Supplementary file 1 [file ijms-24-01307-s001.zip › ijms-2124595-supplementary.pdf]

Table S1. List of Primary Antibodies

Antibody	Company	Catalogue #	Source	Molecular weight	Experiment	Dilution
GRP-78	Cell signaling	3183	Rabbit	78	WB	1:1000
pPERK	biorbyt	orb504147	Rabbit IgG	125	WB	1:1000
PERK	Cell signaling	3192	Rabbit IgG	140	WB	1:1000
peIF2α	Cell signaling	3398	Rabbit IgG	38	WB	1:1000
eIF2α	Cell signaling	5324	Rabbit IgG	38	WB	1:1000
ATF4	Cell signaling	11815	Rabbit IgG	49	WB	1:1000
pIRE1	Invitrogen	PA-105424	Rabbit IgG	110	WB	1:500
IRE1	Cell signaling	3294	Rabbit IgG	130	WB	1:1000
pASK1	Invitrogen	PA5-105027	Rabbit	170	WB	1:1000
pJNK	Cell signaling	9251	Rabbit	46-54	WB	1:1000
JNK	Cell signaling	9252	Rabbit	46-54	WB	1:1000
pP38MAPK	Cell signaling	4511	Rabbit IgG	43	WB	1:1000
P38MAPK	Cell signaling	9212	Rabbit	40	WB	1:1000
PUMA	Cell signaling	12450	Rabbit IgG	23	WB	1:1000
Bax	Cell signaling	5023	Rabbit IgG	20	WB	1:1000
Cleaved Caspase 3	Cell signaling	9664	Rabbit	19	WB	1:1000
Synaptophysin	SantaCruz	SC17750	Mouse	38	WB	1:1000
iNOS	Cell signaling	13120	Rabbit	130	WB	1:1000
β Actin	Cell signaling	5125	Rabbit IgG	45	WB	1:3000
NeuN	Cell signaling	24307	Rabbit IgG	46-55	WB, IHC	1:1000, 1:500
CHOP	Cell signaling	2895	Mouse IgG2a	27	WB, IHC	1:1000, 1:50
Synaptophysin	Cell signaling	36406	Rabbit	38	IHC	1:400
TUNEL	Promega	G7131 &G7132				

Table S1. List of Secondary Antibodies

Antibody	Company	Catalogue #	Experiment	Dilution
Anti-rabbit IgG, HRP-linked antibody	Cell signaling	7074	WB	1:1000
Anti-mouse IgG, HRP-linked antibody	Cell signaling	7076	WB	1:1000
Rabbit Ig, biotinylated	Vector lab.	PK-6101	IHC	1:200
Mouse Ig, biotinylated	Vector lab.	PK-6102	IHC	1:50
